# Remarkable Diversity of *Escherichia coli* Carrying *mcr-1* from Hospital Sewage with the Identification of Two New *mcr-1* Variants

**DOI:** 10.3389/fmicb.2017.02094

**Published:** 2017-10-25

**Authors:** Feifei Zhao, Yu Feng, Xiaoju Lü, Alan McNally, Zhiyong Zong

**Affiliations:** ^1^Center of Infectious Diseases, West China Hospital, Sichuan University, Chengdu, China; ^2^Division of Infectious Diseases, State Key Laboratory of Biotherapy, Chengdu, China; ^3^Institute of Microbiology and Infection, College of Medical and Dental Sciences, University of Birmingham, Birmingham, United Kingdom; ^4^Department of Infection Control, West China Hospital, Sichuan University, Chengdu, China

**Keywords:** colistin resistance, *mcr-1*, sewage, *Escherichia coli*, plasmid

## Abstract

The plasmid-borne colistin-resistant gene *mcr-1* has rapidly become a worldwide public health concern. This study aims to determine the host bacterial strains, plasmids, and genetic contexts of *mcr-1* in hospital sewage. A 1-ml hospital sewage sample was cultured. Colistin-resistant bacterial colonies were selected on agar plates and were subjected to whole genome sequencing and subsequent analysis. The transfer of *mcr-1* between bacterial strains was tested using conjugation. New variants of *mcr-1* were cloned to test the impact of variations on the function of *mcr-1*. Plasmids carrying *mcr-1* were retrieved from GenBank for comparison based on concatenated backbone genes. In the sewage sample, we observed that *mcr-1* was located in various genetic contexts on the chromosome, or plasmids of four different replicon types (IncHI2, IncI2, IncP, and IncX4), in *Klebsiella pneumoniae, Kluyvera* spp. and seven *Escherichia coli* strains of six different sequence types (ST10, ST34, ST48, ST1196, ST7086, and ST7087). We also identified two new variants of *mcr-1, mcr-1.4* and *mcr-1.7*, both of which encode an amino acid variation from *mcr-1*. *mcr-1*-carrying IncX4 plasmids, which have a global distribution across the *Enterobacteriaceae*, are the result of global dissemination of a single common plasmid, while IncI2 *mcr-1* plasmids appear to acquire *mcr-1* in multiple events. In conclusion, the unprecedented remarkable diversity of species, strains, plasmids, and genetic contexts carrying *mcr-1* present in a single sewage sample from a single healthcare site highlights the continued evolution and dynamic transmission of *mcr-1* in healthcare-associated environments.

## Introduction

Colistin (also known as polymycin E) is an antibiotic and has long been one of the last resort treatments for infections caused by multi-drug resistant Gram-negative bacteria. However, bacterial strains that acquired resistance to colistin resistance have emerged worldwide (Olaitan et al., 2014). The mechanisms mediating resistance to colistin are mainly due to mutations or insertions in the chromosomal genes such as the *phoP-Q* and *pmrA-B* and *ccrA-B* two-component systems and the regulator gene *mgrB* (Olaitan et al., 2014). A plasmid-borne colistin resistance gene, *mcr-1*, has recently been found in *Escherichia coli* and in a lesser extent *Klebsiella pneumonia* (Liu et al., 2016). *mcr-1* encodes a phosphoethanolamine (PEA) transferase enzyme that is capable of adding PEA to the lipid A moiety of lipopolysaccharides (LPSs), the initial target of colistin (Liu et al., 2016). Besides *E. coli* and *K. pneumonia, mcr-1* has been detected in various species of the *Enterobacteriaceae* in many countries (Schwarz and Johnson, 2016), imposing an emerging threat for human and animal health. During a screening study for colistin-resistant bacterial isolates in hospital sewage, we found that *mcr-1* genes including two new variants were carried by plasmids of various replicon types in multiple *E. coli* strains.

## Materials and Methods

### Strains

Sewage (1 ml) was collected from the influx of the wastewater treatment plant at West China Hospital in November 2015. West China Hospital is a 5,000-bed tertiary teaching hospital and serves as one of the major referral medical centers in western China. All sewages produced in the hospital were processed in the plant. The sewage sample was mixed with 100 ml brain heart infusion broth (Oxoid, Basingstoke, United Kingdom) in a 500-ml flask. After overnight incubation at 37°C, the culture suspension was diluted to 0.5 McFarland standard and an 100 μl aliquot was plated onto a CHROMAgar Orientation agar plate (CHROMAgar, Paris, France) containing 4 μg/ml colistin and 64 μg/ml linezolid. The plate was then incubated at 37°C overnight. Pink colonies that represent *E. coli* were screened for *mcr-1* as described previously (Liu et al., 2016). Species identification of the colonies was established by partially sequencing the *gyrB* gene as described previously (Yamamoto and Harayama, 1995). MICs of amikacin, cefotaxime, ciprofloxacin, colistin, imipenem, polymycin B, and tigecycline were determined using the microdilution broth method following recommendations of the Clinical Laboratory Standards Institute (CLSI) (CLSI, 2013).

### Cloning of *mcr-1* Variants

The complete coding sequence of *mcr-1.1, mcr-1.4*, and *mcr-1.7* was amplified with primers mcr1-up1 (TGCCGTAATTATCCCACCGT) and mcr1-dw1 (ACCAATCAGCGACCATCAGA) using PrimeSTAR Max DNA Polymerase (Takara, Dalian, China). Amplicons were ligated to the pMD20-T vector using the Mighty TA-cloning kit (Takara). The ligated fragments were transformed into *E. coli* DH5α. pMD20-T::*mcr-1.1*-, pMD20-T::*mcr-1.4*-, or pMD20-T::*mcr-1.7-*containing transformants were selected on LB agar plates containing 2 μg/mL colistin. The presence of *mcr-1.1, mcr-1.4*, or *mcr-1.7* in transformants was confirmed by PCR and sequencing. MICs of colistin were determined for transformants carrying pMD20-T::*mcr-1.1*, pMD20-T::*mcr-1.4*, or pMD20-T::*mcr-1.7* using the broth microdilution method (CLSI, 2013).

### Strain Typing

Pulsed-field gel electrophoresis (PFGE) was performed using the protocol for non-O157 *E. coli* of PulseNet International^1^. *E. coli* strains were assigned to the phylogenetic groups A, B1, B2, and D using PCR as described previously (Clermont et al., 2000).

### Conjugation

Conjugation experiments were carried out in BHI broth and on filter. The azide-resistant *E. coli* strain J53 was used as the recipient and transconjugants were selected on LB agar plates containing 2 μg/ml colistin plus 150 μg/ml sodium azide. The presence of *mcr-1.1, mcr-1.4*, or *mcr-1.7* in transconjugants was confirmed using PCR and sequencing.

### Genome Sequencing and Analysis

Genomic DNA was prepared using the QIAamp DNA Mini Kit (Qiagen, Hilden, Germany). Purified DNA was 150-bp paired-end whole genome sequenced to around 200× coverage using the HiSeq X10 Sequencer (Illumina, San Diego, CA, United States). Reads were *de novo* assembled into contigs using SPAdes (Bankevich et al., 2012). In addition, strain WCHEC1613 was also sequenced using the long-read PacBio RSII Sequencer (Pacific Biosciences, Menlo Park, CA, United States). The assembly was initially built from the PacBio reads alone using program Canu (Koren et al., 2017) with default settings. To obtain high-quality reads for correction, the Illumina reads were trimmed using Trimmomatic (Bolger et al., 2014) with 3, 25, and 50 as the size of sliding window, threshold of mean quality, and minimum length of reads, respectively. The filtered reads were then mapped against the initial assembly to obtain a coordinate sorted BAM file and subsequently a filtered VCF file (minDP10 and minQ30) using Smalt^2^ (version 0.7.4), SAMtools (version 1.3.1) (Li et al., 2009), and VCFtools (version 0.1.14) (Danecek et al., 2011). The final assembly of WCHEC1613 was created by correcting SNPs and indels from the BAM file using PacBio-utilities^3^.

Sequence type (ST) was assigned using the genomic sequence to query the Enterobase database^4^. Antimicrobial resistance genes were predicted using ResFinder^5^. Plasmid sequences carrying *mcr-1* were completely circularized by PCR and Sanger sequencing. For *mcr-1* that was not carried by plasmid, its chromosomal location was confirmed by PCR to link the contig containing *mcr-1* and those containing housekeeping genes belonging to the chromosome.

### Phylogenetic Analyses for IncX4, IncI2, and IncHI2 Plasmids

The sequence of all available IncX4, IncI2, and IncHI2 plasmids regardless of the carriage of *mcr-1* were retrieved from the GenBank (Supplementary Tables 1–3). Genes present on all analyzed IncX4, IncI2, or IncHI2 plasmids were considered as backbone genes, which were identified using OrthoFinder (Emms and Kelly, 2015). Sequences of backbone genes were concatenated and were then aligned to construct a phylogenetic tree for IncX4, IncI2, or IncHI2 plasmids, respectively, using RAxML (Stamatakis, 2014) with a 1,000-bootstrap test.

### Detecting IS*Apl1*-Formed Circular Intermediate

Reverse PCR was performed to amplify *mcr-1* and its surroundings in strain WCHKP_1511 (Zhao et al., 2017), which contains an intact IS*Apl1* upstream of *mcr-1* and an interrupted IS*Apl1* downstream as described previously (Li et al., 2017) and the amplicon was sequenced.

## Results and Discussion

### Seven *mcr-1*-Carrying *E. coli* Strains of Six STs

A total of nine pink colonies (indicative of *E. coli*) were recovered on CHROMAgar agar plates containing 4 μg/ml colistin and 64 μg/ml linezolid from sewage. The nine isolates were designated WCHEC1604, WCHEC1606, WCHEC1609, WCHEC1612, WCHEC1613, WCHEC1614, WCHEC1615, WCHEC1618, and WCHEC1622 here. All of the isolates were identified as *E. coli*, were resistant to colistin (MICs, 4 or 8 μg/ml) and polymyxin B (MIC, 4 μg/ml), and were found to carry *mcr-1* by PCR.

The nine isolates displayed seven different PFGE patterns (data not shown) with two pairs of isolates (WCHEC1612/WCHEC1613, WCHEC1614/WCHEC1615) having identical PFGE patterns, suggesting that the nine isolates belonged to seven strains. Therefore, seven isolates were included for further studies with WCHEC1612 and WCHEC1615 being excluded. All seven strains were susceptible to amikacin, ceftazidime, ciprofloxacin, imipenem, and tigecycline except one strain (WCHEC1604) that was resistant to ciprofloxacin (MIC, 8 μg/ml) and one (WCHEC1609) that was intermediate to ceftazidime (MIC, 8 μg/ml). Nonetheless, all seven strains carried multiple antimicrobial resistant genes (**Table 1**).

**Table 1:** Sequence type (ST), plasmid, and resistance genes of the isolates.

Isolate	ST	ST complex	Plasmid carrying *mcr-1*^a^	Other resistance genes^b^								
				Aminoglycoside	β-lactam	Quinolone	Fosfomycin	MLS^c^	Phenicol	Sulphonamide	Tetracycline	Trimethoprim
WCHEC1604	1196	446	IncX4, 31,229 IncI2, 62,098	*strA, strB, aac(3)-IId, aadA2, aadA1*	*bla*_CTX-M-14_, *bla*_TEM-1b_	*oqxB, oqxA*	*fosA*	*lnu(F)*, *mph(A)*	*cmlA1*, *floR*	*sul1, sul3*	*tet(M)*	*dfrA12*
WCHEC1606	7087	165	IncX4, 33,309	*aac(3)-IId, strA, strB, aadA22*	*bla*_CTX-M-14_, *bla*_TEM-1b_	*qnrS1*		*Mph(A)*	*floR*	*sul2*	*tet(B)*	*dfrA14*
WCHEC1609	10	10		*aph(4)-Ia, aph(3′)-Ia, aac(3)-IVa, aadA2, aadA1*	*bla*_CTX-M-55_, *bla*_TEM-1b_, *bla*_CMY -2_	*oqxA, oqxB*	*fosA*	*mef(B)*	*floR*, *cmlA1*	*sul3*	*tet(A)*	*dfrA12*
WCHEC1613	48	10	IncHI2, IncN 300,307	*aadA1, aph(4)-Ia,* *aac(3)-IVa, aadA2*, *aph(3′)-Ia*, *strA, strB*	*bla*_TEM-1b_, *bla*_CTX-M-14_	*oqxB, oqxA* *qnrS1*	*fosA*	*mph(A)*	*cmlA1*	*sul1, sul2,* *sul3*		*dfrA14*, *dfrA12*
WCHEC1614	34	10		*aph(3′)-Ia, aadA2, aadA1*	*bla*_TEM-1b_			*mef(B)*	*floR*, *cmlA1*	*sul3*	*tet(A)*	*dfrA12*
WCHEC1618	34	10	IncX4, 33,309	*aph(3′)-Ia, aadA2, aadA1*	*bla*_TEM-1b_	*qnrS1*			*cmlA1*, *floR*	*sul3*	*tet(A)*	*dfrA12*
WCHEC1622	7086	155	IncP, 49,897	*strB, strA*	*bla*_TEM-1b_, *bla*_CTX-M-125_	*qnrS1*			*floR*	*sul2*	*tet(A)*	*dfrA14*

*^a^Replicon type and size of plasmids are shown.*

*^b^Resistance genes that were located on plasmids carrying *mcr-1* are underlined.*

*^c^MLS: macrolide, lincosamide, and streptogramin B.*

Draft genome sequences of the seven selected isolates were generated by Illumina whole genome sequencing, which were assembled into 141–242 contigs (83–134 contigs ≥ 1,000 bp in length; *N50*, 97,014–253,501 bp) with a 50.27–50.80% GC content, respectively (**Table 2**). Strain WCHEC1613 was also sequenced using PacBio, which generated 48,400 reads and 451,334,091 bases. A hybrid assembly of the PacBio data with the Illumina reads formed four circular contigs representing one chromosome and three plasmids for strain WCHEC1613.

**Table 2:** General features of the seven genomes.

Strain	ST	Clean reads	Draft genome size (bp)	GC content	No. of contigs	No. of contigs ≥ 1,000 bp	No. of coding sequences	No. of tRNA genes
WCHEC1604	1196	4,567,732	5,413,166	50.27	201	101	5,102	88
WCHEC1606	7087	3,944,022	4,867,654	50.67	141	83	4,545	84
WCHEC1609	10	4,198,787	4,978,999	50.68	242	118	4,664	75
WCHEC1613^a^	48	48,400	5,168,735	50.66	4	4	4,875	90
WCHEC1614	34	4,447,267	4,725,432	50.75	197	117	4,453	81
WCHEC1618	34	5,208,807	4,707,492	50.80	166	99	4,415	82
WCHEC1622	7086	4,093,859	4,910,938	50.65	232	134	4,632	78

*ST, sequence type. ^a^The features of strain WCHEC1613 are from PacBio sequencing.*

The seven strains were belonged to six STs, ST10, ST34, ST48, ST1196, ST7086, and ST7087 with the latter two being new types, which have not been identified before. ST7086 has a single allele (*fumC*) different from ST155, while ST7087 differs from ST165 by one allele (*mdh*). Of note, strains WCHEC1614 and WCHEC1618 belonged to the same ST (ST34) but there were 554 single nucleotide polymorphisms (SNPs) between their genomes, suggesting that the two strains are likely divergent over a manner of years rather than days or weeks (Stoesser et al., 2016). Therefore, in a single sewage sample, we identified seven *E. coli* strains belonging to six different STs. Previous reports of *mcr-1* gene carriage in *E. coli* have identified a similarly diverse range of STs carrying the resistance gene (Kong et al., 2016; El Garch et al., 2017; Quan et al., 2017; Wang et al., 2017), though none have reported such diversity in a single confined sample type. This indicates that the dissemination of *mcr-1* is not due to expansions of high-risk clones, but rather that *mcr-1* is frequently being acquired across the *E. coli* population in multiple independent events. In the same hospital sewage sample, there were also two blue colonies that were found to carry *mcr-1*. The two blue colonies were identified as *Kluyvera* spp. and *K. pneumoniae* and have been reported elsewhere (Zhao and Zong, 2016; Zhao et al., 2017). Nonetheless, hospital sewage accumulates high density of bacteria, while antibiotics, disinfectants, and their metabolic products are disposed of into hospital sewage and impose selection pressure in favor of the existence of antimicrobial resistant bacteria (Kummerer, 2004), which might explain the diversity of *mcr-1*-carrying isolates seen here.

### Two New *mcr-1* Variants

Sequencing the whole coding sequence of *mcr-1* revealed the original *mcr-1*, designated *mcr-1.1* here, in eight isolates, one of which (strain WCHEC1604) contained two *mcr-1* variants including *mcr-1.1* and a new variant, designated *mcr-1.7* here. The remaining strain, WCHEC1606, had another new variant of *mcr-1.1*, designated *mcr-1.4* here. Both *mcr-1.4* and *mcr-1.7* have a single nucleotide substitution (G1318T and G643A, respectively) compared to *mcr-1.1*, resulting in an amino acid substitution (G1318T and A215T, respectively). In addition to *mcr-1.4* and *mcr-1.7*, there are seven variants of *mcr-1* in GenBank. Four variants have a single amino acid variation from *mcr-1.1*, while the remaining three have two or three amino acid substitutions compared with *mcr-1.1* (**Table 3**).

**Table 3:** *mcr-1* variants.

	Accession numbers	Nucleotide mutations	Amino acid variations	Locations of amino acid variations	Host strain	Source	Country	Year	Reference
*mcr-1.2*	KX236309	T8A	Q3L	TM domain	*K. pneumoniae*	Human rectal swab	Italy	2014	Di Pilato et al., 2016
*mcr-1.3*	KU934208	G111A, G112A	I38V	TM domain	*E. coli*	Chicken	China	Unknown	
*mcr-1.4*		G1318T	D439N	α6 unit, PEA transferase domain	*E. coli*	Sewage	China	2015	This study
*mcr-1.5*	KY283125	C1354T	H452Y	Region between α6 and β7 unit, PEA transferase domain	*E. coli*	Human urine	Argentina	2015	
*mcr-1.6*	KY352406	A1263G, A1607G	T215A, R536H	Region prior to β1 unit, η12 unit, PEA transferase domain					
*mcr-1.7*		G643A	A215T	Region prior to β1 unit, PEA transferase domain	*E. coli*	Sewage	China	2015	This study
*mcr-1*, unnamed	MADL0100 0078.1	T933A, C946T, T947C, A967T, T987C, A999G	N311K, L316S, I323F	α4 and β5 unit, PEA transferase domain	*E. coli*	Lizard	Germany (imported from Vietnam)	2013	Unger et al., 2017
*mcr-1*, unnamed 2	MOFD0100 0034.1	C1396A	H466N	Region between β6 and β7 unit, PEA transferase domain	*E. coli*	Chicken	China	2014	
*mcr-1*, unnamed 3	MTKG0100 0192.1	G24C	W8C	TM domain	*E. coli*	Seawater	Brazil	2016	

The *mcr-1* contains a transmembrane domain and a PEA transferase domain with 8α, 12β, and 12η units (Gao et al., 2016). The variations of *mcr-1.4* and *mcr-1.7* occurred in the region prior to the β1 unit and in the α6 unit of the PEA transferase domain, respectively, both of which have been found not to influence the function of *mcr-1* (Gao et al., 2016). MICs of colistin against transformants containing pMD20-T::*mcr-1.4* and pMD20-T::*mcr-1.7* were both 4 μg/ml. Among other *mcr-1.1* variants, only *mcr-1.2* has been characterized at present. MIC of colistin against an *E. coli* transconjugant containing *mcr-1.2* was 8 μg/ml (Di Pilato et al., 2016). Therefore, *mcr-1.2, mcr-1.4*, and *mcr-1.7* have unaltered activity against colistin compared to *mcr-1.1*. The impact of amino acid substitutions seen in other *mcr-1* variants on the function of *mcr-1* remains unclear and warrants further investigations.

### Plasmids Carrying *mcr-1*

We sought to determine whether there are signatures of plasmid dissemination through the *E. coli* population present in our sewage sample. In two strains (WCHEC1609 and WCHEC1614), *mcr-1* was located on the chromosome, while in the remaining five strains, *mcr-1* was carried by a plasmid belonging to four different replicon types including IncHI2, IncI2, IncP, and IncX4 (**Table 1**). Of note, *mcr-1.1* was located on an IncX4 plasmid and *mcr-1*.*7* was on an IncI2 plasmid in strain WCHEC1604. It is remarkable that *mcr-1*-carrying plasmids of four replicon types were found in a single 1 ml sewage sample at one site. All IncI2, IncP, and IncX4 *mcr-1*-carrying plasmids in this study could be transferred by conjugation at a frequency of 10^-5^ to 10^-7^ cells per recipient cell by mating, while the *mcr-1*-carrying IncHI2 plasmid was not.

**FIGURE 1:**
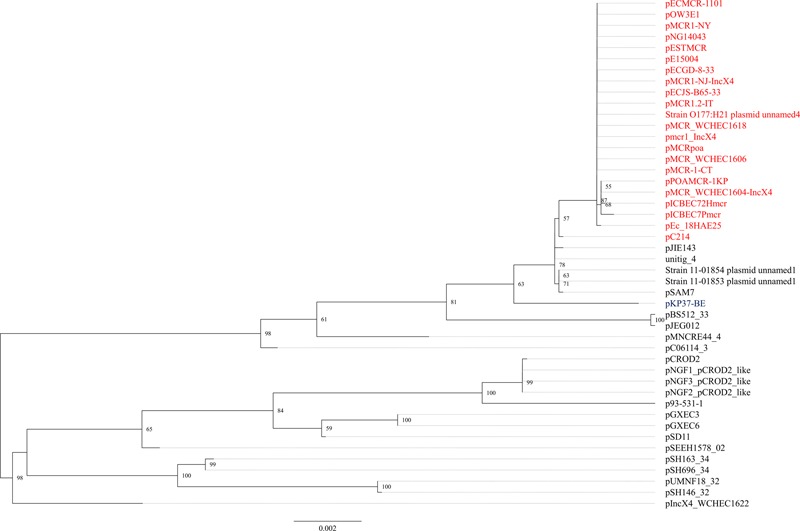
Phylogenetic analysis of IncX4 plasmids. The accession numbers of the plasmids are shown in Supplementary Table 1. Those carrying *mcr-1* are indicated in red, while pKP37-BE carrying *mcr-2* is shown in blue. The tree was constructed using concatenated sequences of 17 genes belonging to the IncX4 backbone.

The *mcr-1.1* gene was carried by IncX4 plasmids in two strains, WCHEC1604 and WCHEC1618, designated pMCR_1604-IncX4 and pMCR_WCHEC1618, respectively. *mcr-1.4* in strain WCHEC1606 was also located on an IncX4 plasmid (designated pMCR_WCHEC1606). pMCR_1604-IncX4 is 31,229 bp in length and is 2,080 bp less than the 33,309-bp pMCR_WCHEC1618 and pMCR_WCHEC1606, which was likely due to homologous recombination between the two copies of the *dnaJ-*containing region. pMCR_WCHEC1606 differed from pMCR_WCHEC1618 by only a single nucleotide substitution, which was the one defining *mcr-1.4*, suggesting that *mcr-1.4* evolved from *mcr-1.1* by a point mutation on the IncX4 plasmid. IncX4 plasmids carrying *mcr-1* have been found in *E. coli* or *K. pneumoniae* strains in Africa (South Africa), Asia (China), Europe (Estonia, Italy, Netherlands, and Switzerland), and North (United States) and South America (Brazil), suggesting a global distribution. Phylogenetic analysis based on all 17 backbone genes of IncX4 plasmids (Supplementary Table 1) revealed that all *mcr-1*-carrying IncX4 plasmids formed a clade with several non-*mcr-1*-carrying IncX4 plasmids (**Figure 1**), suggesting that the *mcr-1*-carrying IncX4 plasmids were likely from a common ancestor and the acquisition of *mcr-1* onto the IncX4 backbone occurred recently. By contrast, *mcr-2*-carrying IncX4 plasmid pKP37-BE was distinct from *mcr-1*-carrying IncX4 plasmids (**Figure 1**).

**FIGURE 2:**
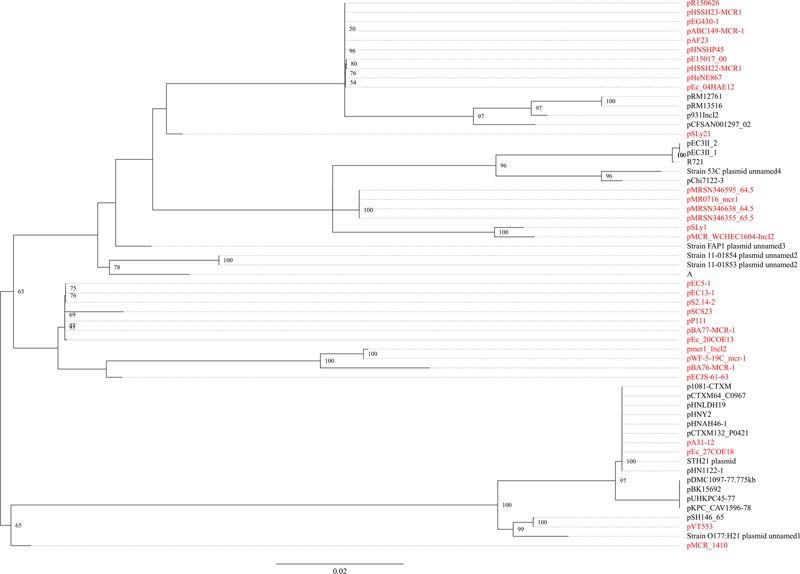
Phylogenetic analysis of IncI2 plasmids. The accession numbers of the plasmids are shown in Supplementary Table 2. Those carrying *mcr-1* are indicated in red. The tree was constructed using concatenated sequences of 27 genes belonging to the IncI2 backbone.

The *mcr-1.7* in strain WCHEC1604 was carried by a 62,098-bp IncI2 plasmid, designated pMCR_1604-IncI2 here. Phylogenetic analysis based on all 27 backbone genes of IncI2 plasmids (Supplementary Table 2) revealed that *mcr-1*-carrying plasmids belonged to a number of clades and mixed with plasmids without *mcr-1*, suggesting that IncI2 plasmids were likely to have acquired *mcr-1* in multiple events rather than a single plasmid expansion into different strains (**Figure 2**). pMCR_1604-IncI2 was most closely related (99% identity and 93% coverage) to pECJS-61-63 (GenBank accession no. KX254342) in an *E. coli* isolated from a pig in China and it is likely that *mcr-1.7* evolved from *mcr-1.1* on an IncI2 plasmid.

**FIGURE 3:**
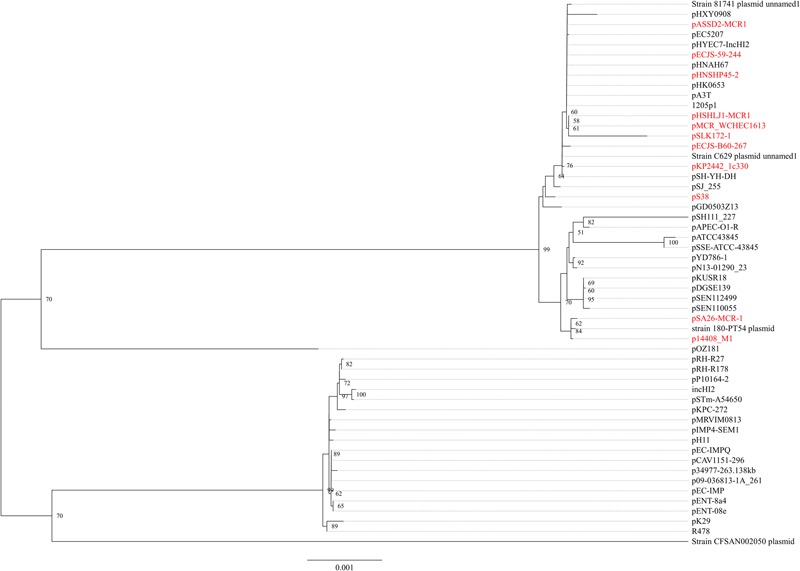
Phylogenetic analysis of IncHI2 plasmids. The accession numbers of the plasmids are shown in Supplementary Table 3. Those carrying *mcr-1* are indicated in red. The tree was constructed using concatenated sequences of 27 genes belonging to the IncI2 backbone.

In strain WCHEC1613, *mcr-1.1* was carried by a 300-kb large plasmid (designated pMCR_WCHEC1613) containing both IncHI2 and IncN replicons. pMCR_WCHEC1613 was most closely related (83% coverage and 99% identity) to the IncHI2 plasmid pHNSHP45-2 (GenBank accession no. KU341381) carrying *mcr-1.1* from *E. coli* strain SHP45 in China (Liu et al., 2016). In addition, a large part (94,689 bp; positions 73,442–170,084) of pMCR_WCHEC1613 was nearly identical to the counterpart of a plasmid containing both IncFIB and IncN replicons, pMR0516mcr (GenBank accession no. KX276657), carrying *mcr-1.1* from *E. coli* in United States (McGann et al., 2016). It is likely that pMCR_WCHEC1613 was formed by the fusion of two plasmids, which contain IncHI2 and IncN replicon, respectively. Phylogenetic analysis based on all 33 backbones genes of IncHI2 plasmids (Supplementary Table 3) revealed that all *mcr-1*-carrying IncHI2 plasmids were clustered together with a few non-*mcr-1*-carrying IncHI2 plasmids (**Figure 3**), suggesting that the *mcr-1*-carrying IncHI2 plasmids arose from a common ancestor and the acquisition of *mcr-1* onto the IncHI2 backbone occurred recently.

**FIGURE 4:**
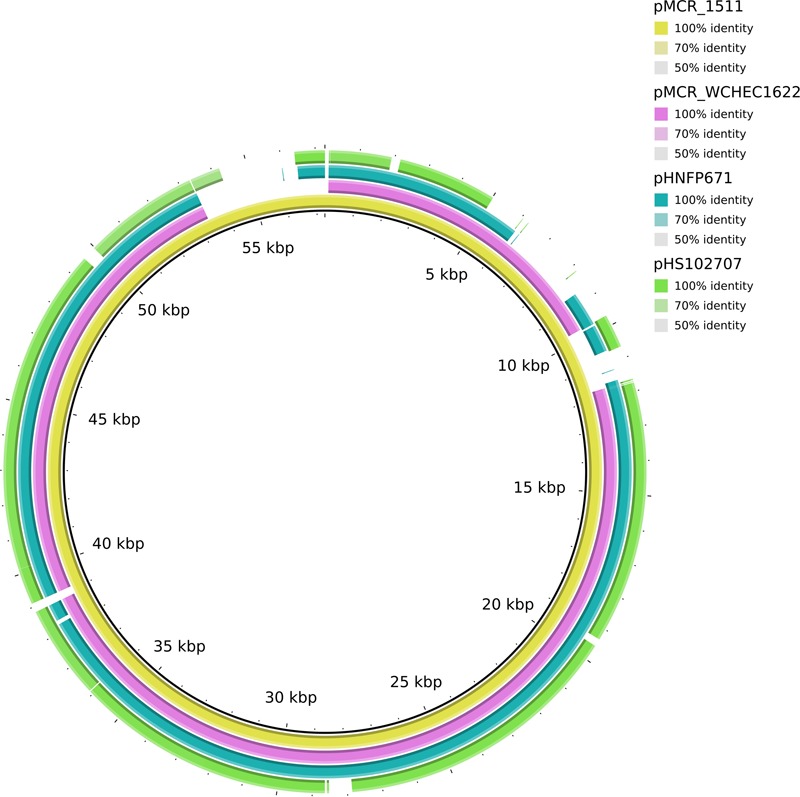
Alignment of pMCR_WCHEC1622 and closely related IncP plasmids. pMCR_1511 (Zhao et al., 2017) was used as a reference. The alignment is a pairwise BLASTn alignment performed using BRIG. pMCR_1511, pMCR_WCHEC1622, pHNFP671 (GenBank accession no. KP324830), pHS102707 (GenBank accession no. KF701335), pMCR16_P053 (GenBank accession no. KY352406), and pJJ1886_4 (GenBank accession no. CP006788) belong to a new unnamed clade of IncP plasmids (Zhao et al., 2017).

In WCHEC1622, *mcr-1.1* was carried by a 49,897-bp IncP plasmid, designated pMCR_WCHEC1622, which was almost identical (100% coverage and 99% identity) to pMCR_1511 (**Figure 4**), an IncP plasmid recovered from *K. pneumoniae* in the same sewage sample (Zhao et al., 2017). Like pMCR_1511, pMCR_WCHEC1622 belongs to a new clade of IncP (Zhao et al., 2017). As IncP plasmids are broad-host-range, the carriage of *mcr-1* on IncP plasmids has the potential to mediate the dissemination of *mcr-1* from the *Enterobacteriaceae* to other bacterial species.

### Genetic Contexts of *mcr-1*

**FIGURE 5:**
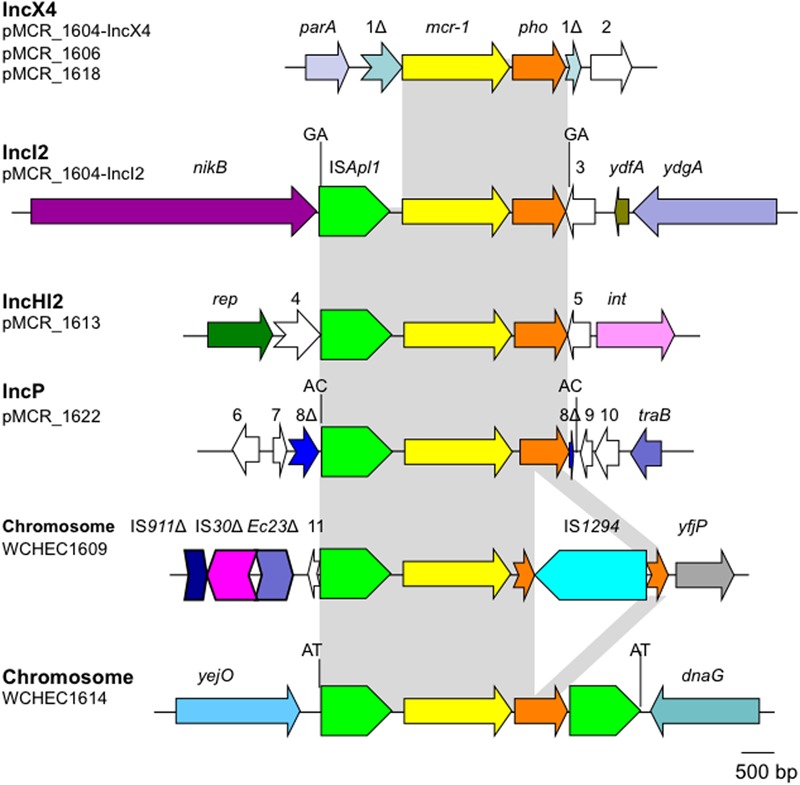
Genetic contexts of *mcr-1* in the seven *E. coli* strains. orfs that encode hypothetical proteins with unknown function are indicated in white except that those interrupted by the element carrying *mcr-1*. Orfs are indicated with numbers, 1–11. Δ represents interrupted or truncated. Other genes are *dnaG* (encoding a DNA primase), *int* (encoding a recombinase), *parA* (encoding partition), *nikB* (encoding a relaxase of the plasmid), *rep* (encoding the replication protein), *traB* (encoding a conjugative protein), *ydfA* (a transcriptional regulator), *ydgA* (encoding a DNA topoisomerase III), *yejO* (an outer membrane β-barrel domain-containing protein), and *yfjP* (encoding a 50S ribosome-binding GTPase). The 2-bp direct repeat (DR) is shown if it is present.

**FIGURE 6:**
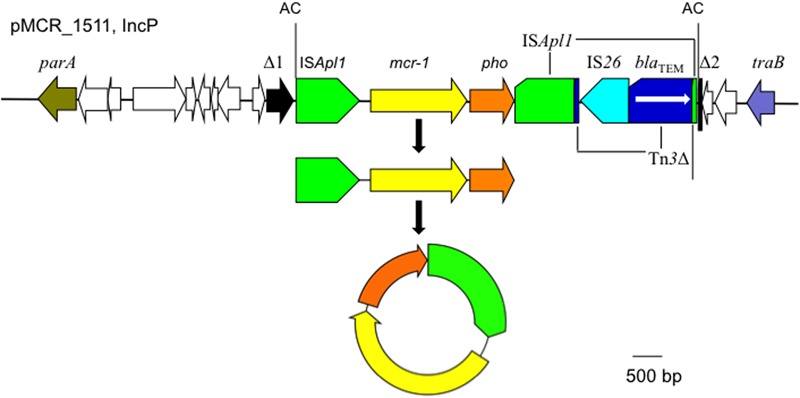
The circular intermediate formed by IS*Apl1*. Genetic context of *mcr-1* on the IncP plasmid pMCR_1511 is shown. Orfs that encode hypothetical proteins with unknown function are indicated in white except that the one disrupted by the IS*Apl1*-formed composite transposon on pMCR_1511 is shown in black (Δ1 and Δ2). The 2-bp direct repeat (AC) abutting the IS*Apl1*-formed composite transposon on pMCR_1511 is shown. On pMCR_1511, the IS*Apl1* downstream of *mcr-1* was interrupted by Tn*3*, which was interrupted by IS*26*.

The *mcr-1* has been typically seen in three types of genetic contexts, i.e., *mcr-1*-*pho*, IS*Apl1*-*mcr-1*-*pho*, and IS*Apl1*-*mcr-1*-*pho*-IS*Apl1* (Gao et al., 2016; Snesrud et al., 2016; Li et al., 2017) with *pho* referring to a gene (also called *pap* in some publications) encoding a putative phosphoesterase and IS*Apl1* being an insertion sequence of the IS*30* family. All three types of the *mcr-1* genetic context were seen in the seven *E. coli* strains here (**Figure 5**). Two copies of IS*Apl1* bracketing *mcr-1* and *pho* (IS*Apl1*-*mcr-1*-*pho*-IS*Apl1*) could form a composite transposon termed Tn*6330*, which is able to generate a circular intermediate (IS*Apl1*-*mcr-1*-*pho*) by excision from a plasmid and the intermediate could then insert into another plasmid (Li et al., 2017) and possibly could also insert into chromosome. A previous study (Li et al., 2017) found that the circular intermediate is formed by the IS*Apl1* downstream of *mcr-1*. However, in this study, the IS*Apl1*-*mcr-1*-*pho* circular intermediate was obtained from pMCR_1511, in which the IS*Apl1* downstream of *mcr-1* was interrupted (**Figure 6**). This suggests that the IS*Apl1* upstream of *mcr-1* participated in the formation of the IS*Apl1*-*mcr-1*-*pho* circular intermediate by its own or via recombination with the IS*Apl1* downstream of *mcr-1*.

## Conclusion

Therefore, in a single (1 mL) hospital sewage sample, we observed multiple *Enterobacteriaceae* species, multiple strains of *E. coli*, multiple plasmids, and multiple genetic contexts carrying multiple variants of *mcr-1*. This suggests that *mcr-1* is undergoing rapid evolution within healthcare environments and is being rapidly disseminated across plasmids, strains, and species.

Nucleotide sequence accession numbers: Reads and the Whole Genome Shotgun Sequencing project of *E. coli* strain WCHEC1604, WCHEC1606, WCHEC1609, WCHEC1613, WCHEC1614, WCHEC1618, and WCHEC1622 have been deposited into DDBJ/EMBL/GenBank under accession MUWZ00000000, MSRB00000000, MSQX00000000, CP019213, MSQY00000000, MSQZ00000000, and MSRA00000000, respectively. The sequence of pMCR_1604-IncX4, pMCR_1604-IncI2, pMCR_WCHEC1606, pMCR_WCHEC1613, pMCR_WCHEC1618, and pMCR_WCHEC1622 has been deposited into DDBJ/EMBL/GenBank under accession numbers KY582848, KY829117, KY463451, CP019214, KY463454, and KY463452, respectively.

## Author Contributions

ZZ: Designed the experiments, analyzed the data, and wrote the manuscript. FZ and YF: Performed the experiments and analyzed the data. XL and AM: Contributed to analyzing the data and co-wrote the manuscript.

## Conflict of Interest Statement

The authors declare that the research was conducted in the absence of any commercial or financial relationships that could be construed as a potential conflict of interest.

## Footnotes

1 http://www.pulsenetinternational.org/protocols/pfge/

2 https://www.sanger.ac.uk/resources/software/smalt/

3 https://github.com/douglasgscofield/PacBio-utilities

4 http://enterobase.warwick.ac.uk/

5 http://genomicepidemiology.org/
