# Supplementary material for: Remarkable Diversity of Escherichia coli Carrying mcr-1 from Hospital Sewage with the Identification of Two New mcr-1 Variants
Source: Front Microbiol. 2017 Oct 25;8:2094. doi: 10.3389/fmicb.2017.02094 (PMC5660977; doi:10.3389/fmicb.2017.02094)
Supplement: Supplementary file 1 [file Data_Sheet_1.DOCX]

**Supplementary data**

Table 1. The accession numbers of IncX4 plasmids.

| Plasmid*^a^* | Accession number |
| --- | --- |
| With *mcr-1* |  |
| pICBEC72Hmcr | CP015977 |
| pESTMCR | KU743383 |
| pmcr1_IncX4 | KU761327 |
| pECJS-B65-33 | KX084392 |
| pOW3E1 | KX129783 |
| pMCR1.2-IT | KX236309 |
| pECGD-8-33 | KX254343 |
| pMCR1-NJ-IncX4 | KX447768 |
| pECMCR-1101 | KX570748 |
| pE15004 | KX772777 |
| pEc_18HAE25 | KY012276 |
| pC214 | KY120363 |
| pNG14043 | KY120364 |
| Strain O177:H21 plasmid unnamed4 | NZ_CP016550 |
| pICBEC7Pmcr | NZ_CP017246 |
| pPOAMCR-1KP | NZ_CM008162 |
| unitig_4 | NZ_CP021735 |
| pMCR1-NY | NZ_CP019908 |
| pMCRpoa | NZ_CM007714 |
| pMCR-1-CT | NZ_CP018773 |
| **pMCR_WCHEC1604-IncX4** | **KY582848** |
| **pMCR_WCHEC1606** | **KY463451** |
| **pMCR_WCHEC1618** | **KY463454** |
|  |  |
| With *mcr-2* |  |
| pKP37-BE | LT598652 |
|  |  |
| Without *mcr* |  |
| pBS512_33 | NC_010657 |
| pCROD2 | NC_013718 |
| pSH696_34 | NC_019128 |
| pSH146_32 | NC_019129 |
| pSH163_34 | NC_019130 |
| pJEG012 | NC_021079 |
| pSEEH1578_02 | NC_021841 |
| pSAM7 | NC_022105 |
| pUMNF18_32 | NZ_AGTD01000006 |
| pNGF2_pCROD2_like | NZ_CM004486 |
| pNGF3_pCROD2_like | NZ_CM004488 |
| pMNCRE44_4 | NZ_CP010880 |
| Strain 11-01853 plasmid unnamed1 | NZ_CP011290 |
| Strain 11-01854 plasmid unnamed1 | NZ_CP011293 |
| pNGF1_pCROD2_like | NZ_CP016008 |
| pC06114_3 | CP016037 |
| pJIE143 | JN194214 |
| pSD11 | KM212169 |
| pGXEC3 | KM580532 |
| pGXEC6 | KM580533 |
| p93-531-1 | KT754165 |
| **pIncX4_WCHEC1622** | KY652381 |

*^a^*Plasmids that were identified in the present study are shown in bold.

Table 2. The accession numbers of IncI2 plasmids.

| Plasmid*^a^* | Accession number |
| --- | --- |
| With *mcr-1* |  |
| pEC5-1 | CP016185 |
| pEC13-1 | CP016186 |
| pS2.14-2 | CP016187 |
| pHNSHP45 | KP347127 |
| pmcr1_IncI2 | KU761326 |
| pVT553 | KU870627 |
| pHeNE867 | KU934208 |
| pSCS23 | KU934209 |
| pABC149-MCR-1 | KX013538 |
| pBA77-MCR-1 | KX013539 |
| pBA76-MCR-1 | KX013540 |
| pAF23 | KX032519 |
| pA31-12 | KX034083 |
| pECJS-61-63 | KX084393 |
| pWF-5-19C_mcr-1 | KX505142 |
| pEc_04HAE12 | KX592672 |
| pE15017_00 | KX772778 |
| pHSSH22-MCR1 | KX856067 |
| pHSSH23-MCR1 | KX856068 |
| pEc_20COE13 | KY012274 |
| pEc_27COE18 | KY012275 |
| pP111 | KY120365 |
| pR150626 | KY120366 |
| pEG430-1 | LT174530 |
| pSLy1 | NZ-CP015913 |
| pSLy21 | NZ-CP016405 |
| pMR0716_mcr1 | NZ-CP018106 |
| pMRSN346595_64.5 | NZ-CP018112 |
| pMRSN346638_64.5 | NZ-CP018118 |
| pMRSN346355_65.5 | NZ-CP018124 |
| pMCR_1410 | KU922754 |
| **pMCR_1604-IncI2** |  |
|  |  |
| Without *mcr* |  |
| STH21 plasmid | LN623683 |
| R721 | NC-002525 |
| pChi7122-3 | NC-019039 |
| pSH146_65 | NC-019115 |
| pHN1122-1 | NC-020270 |
| pBK15692 | NC-022520 |
| pRM13516 | NZ-CP006264 |
| pRM12761 | NZ-CP007134 |
| Strain FAP1 plasmid unnamed3 | NZ-CP009581 |
| A | NZ-CP010220 |
| Strain 11-01853 plasmid unnamed2 | NZ-CP011291 |
| Strain 11-01854 plasmid unnamed2 | NZ-CP011294 |
| pKPC_CAV1596-78 | NZ-CP011645 |
| pDMC1097-77.775kb | NZ-CP011978 |
| Strain O177:H21 plasmid unnamed1 | NZ-CP016547 |
| pCFSAN001297_02 | NZ-CP019199 |
| pUHKPC45-77 | NZ-JMSX01000003 |
| Strain 53C plasmid unnamed4 | NZ-JXMX01000008 |

*^a^*Plasmid that was identified in the present study is shown in bold.

Table 3. The accession numbers of IncHI2 plasmids.

| Plasmid*^a^* | Accession number |
| --- | --- |
| With *mcr-1* |  |
| pHNSHP45-2 | KU341381 |
| **pMCR_WCHEC1613** | NZ_CP019214 |
| pSLK172-1 | NZ_CP017632 |
| pECJS-B60-267 | KX254341 |
| pS38 | KX129782 |
| pECJS-59-244 | KX084394 |
| pSA26-MCR-1 | KU743384 |
| pHSHLJ1-MCR1 | KX856066 |
| p14408_M1 | LT599829 |
| pASSD2-MCR1 | KX856065 |
| pKP2442_1c330 | KX434879 |
|  |  |
| Without *mcr* |  |
| pGD0503Z13 | KR653209 |
| pSSE-ATCC-43845 | NZ_CP016838 |
| pATCC43845 | CP019195 |
| pIMP4-SEM1 | KX810825 |
| pEC-IMPQ | NC_012556 |
| pEC-IMP | NC_012555 |
| pP10164-2 | KX710093 |
| pMRVIM0813 | KP975077 |
| pSTm-A54650 | NC_024983 |
| incHI2 | LN794248 |
| pRH-R27 | LN555650 |
| pCAV1151-296 | NZ_CP011601 |
| pH11 | CP013215 |
| pKPC-272 | NZ_CP008825 |
| pOZ181 | NZ_CP016764 |
| R478 | NC_005211 |
| pEC5207 | KT347600 |
| pK29 | NC_010870 |
| p34977-263.138kb | NZ_CP012170 |
| p09-036813-1A_261 | NZ_CP016526 |
| pSJ_255 | NZ_CP011062 |
| pENT-8a4 | NZ_CP008899 |
| pA3T | KX421096 |
| pHXY0908 | KM877269 |
| 1205p1 | NZ_CP012141 |
| pHK0653 | KT334335 |
| pAPEC-O1-R | NC_009838 |
| strain 180-PT54 plasmid | NZ_CP015833 |
| pN13-01290_23 | NZ_CP012931 |
| Strain 81741 plasmid unnamed1 | NZ_CP019443 |
| pHNAH67 | KX246266 |
| pSH111_227 | NC_019114 |
| pSH-YH-DH | KX129949 |
| pYD786-1 | KU254578 |
| pHYEC7-IncHI2 | KX518743 |
| pRH-R178 | HG530658 |
| pSEN110055 | KM396300 |
| pSEN112499 | KM396299 |
| pDGSE139 | KM198330 |
| pKUSR18 | KM396298 |
| Strain C629 plasmid unnamed1 | NZ_CP015725 |
| pENT-08e | NZ_CP008906 |
| Strain CFSAN002050 plasmid | NC_021845 |

*^a^*Plasmid that was identified in the present study is shown in bold.
